# Reconstitution of a minimal motility system based on *Spiroplasma* swimming by two bacterial actins in a synthetic minimal bacterium

**DOI:** 10.1126/sciadv.abo7490

**Published:** 2022-11-30

**Authors:** Hana Kiyama, Shigeyuki Kakizawa, Yuya Sasajima, Yuhei O. Tahara, Makoto Miyata

**Affiliations:** ^1^ Graduate School of Science, Osaka City University, 3-3-138 Sugimoto, Sumiyoshi-ku, Osaka 558-8585, Japan.; ^2^ Graduate School of Science, Osaka Metropolitan University, 3-3-138 Sugimoto, Sumiyoshi-ku, Osaka 558-8585, Japan.; ^3^ Bioproduction Research Institute, National Institute of Advanced Industrial Science and Technology, Tsukuba, Japan.; ^4^ The OCU Advanced Research Institute for Natural Science and Technology (OCARINA), Osaka Metropolitan University, 3-3-138 Sugimoto, Sumiyoshi-ku, Osaka 558-8585, Japan.

## Abstract

Motility is one of the most important features of life, but its evolutionary origin remains unknown. In this study, we focused on *Spiroplasma*, commensal, or parasitic bacteria. They swim by switching the helicity of a ribbon-like cytoskeleton that comprises six proteins, each of which evolved from a nucleosidase and bacterial actin called MreB. We expressed these proteins in a synthetic, nonmotile minimal bacterium, JCVI-syn3B, whose reduced genome was computer-designed and chemically synthesized. The synthetic bacterium exhibited swimming motility with features characteristic of *Spiroplasma* swimming. Moreover, combinations of *Spiroplasma* MreB4-MreB5 and MreB1-MreB5 produced a helical cell shape and swimming. These results suggest that the swimming originated from the differentiation and coupling of bacterial actins, and we obtained a minimal system for motility of the synthetic bacterium.

## Teaser

Two bacterial actins provide cell helicity and swimming capabilities to a synthetic minimal bacterium.

## INTRODUCTION

Motility is observed in various phyla and is arguably one of the major determinants of survival. If we focus on the force-generating units of cell motility, then all cell motilities reported to date can be classified into 18 mechanisms (*1*). In general, the direct evolutionary ancestor of the individual mechanisms cannot be identified probably because several of these have been in existence for a long time. However, it is possible to discuss their origin and evolution. Cell motility is considered to originate from the rather small movements of housekeeping proteins, for example, adenosine 5′-triphosphate (ATP) synthase, helicase, actin, and tubulin (*1*). These movements were amplified and transmitted to the cell outside possibly because of the accumulation of mutations. However, this process has not yet been experimentally demonstrated. Class Mollicutes are parasitic or commensal bacteria that are characterized by a small genome (*2*, *3*). There are three unique motility mechanisms in Mollicutes (*4*–*6*). It is likely that when the phylum Firmicutes evolved to stop peptidoglycan synthesis, they also stopped flagellar motility, which depends on the peptidoglycan layer, and then acquired unique motility (*1*, *5*). In one of the three types of motilities, when *Spiroplasma* swims, they thrust water by switching the handedness of their helicity (*4*, *7*–*9*). These schemes are completely different from those of the spirochete, a group of bacteria with helical cells. The helical shape of *Spiroplasma* is likely determined by a ribbon-like cytoskeleton, which comprises fibril protein evolved from nucleosidases (*10*–*12*) and five classes of *Spiroplasma* MreBs evolved from MreB, the bacterial actin (*12*–*15*). Here, we refer to *Spiroplasma* MreBs as SMreBs because they are distantly related to MreBs found in walled bacteria (*13*, *16*, *17*). The helicity of the ribbon is determined by the fibril protein, but the mechanism of helicity switching is unknown.

The synthetic bacterium JCVI-syn3.0 was established by J. Craig Venter Institute (JCVI) in 2016 as a combination of a cell of *Mycoplasma capricolum* and a genome designed on the basis of *Mycoplasma mycoides* (*18*). Both *Mycoplasma* species belong to the *Spiroplasma* clade, one of four Mollicutes clades (*2*). JCVI-syn3.0 has the genome of minimal gene set and a fast growth rate, which is beneficial for genome manipulation, roughly spherical morphology, and no motility (*18*, *19*). JCVI-syn3B (syn3B) has 19 genes returned from *M. mycoides* for better growth (*19*, *20*). In this study, we reconstituted *Spiroplasma* swimming in syn3B by adding seven genes and identified the minimal gene set for *Spiroplasma* cell helicity and swimming.

## RESULTS

### Reconstitution of *Spiroplasma* swimming in syn3B

**Fig. 1.**
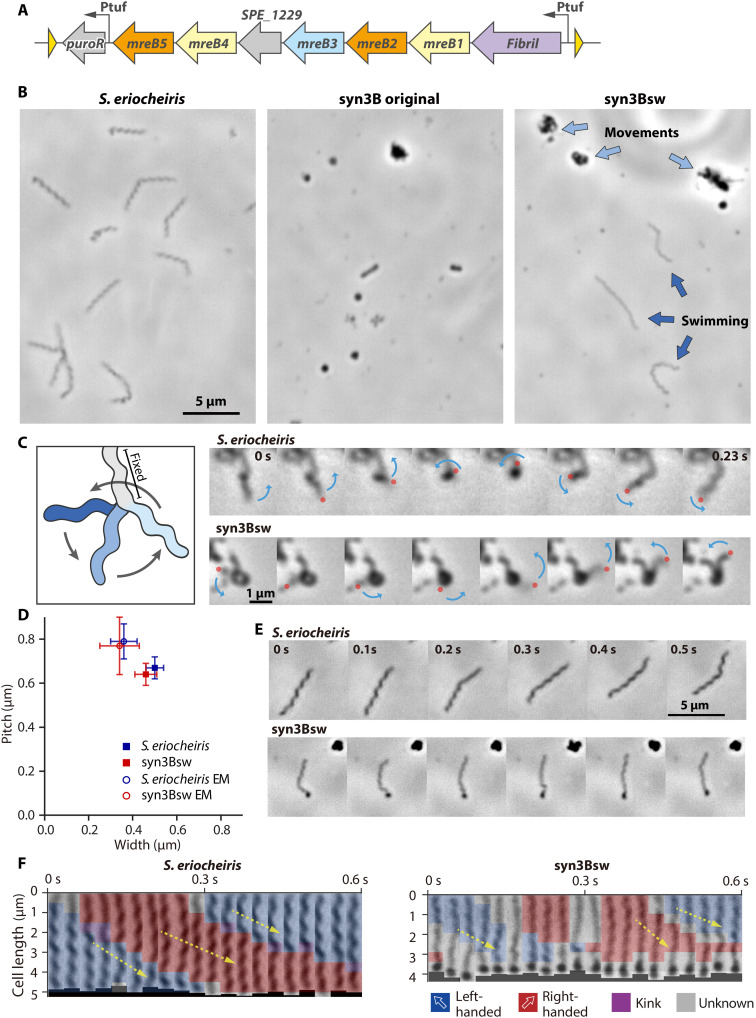
Reconstitution of *Spiroplasma* swimming in syn3B by expressing seven genes. (**A**) A DNA fragment transferred between two *loxP* sites of syn3B, including seven genes from *Spiroplasma* and a puromycin resistance gene, “*puroR.*” A nonannotated gene, *SPE_1229*, is indicated by a gray arrow. Ptuf and *loxP* sites are denoted by black and yellow triangles, respectively. (**B**) Field cell images of three strains indicated on the top. In syn3Bsw, DNA fragment illustrated in (A) is inserted into the genome by Cre*/loxP* system. Cells with characteristic morphology and movements are marked by blue arrows. The cells were observed by phase contrast microscopy. (**C**) Rotational behaviors of freely moving parts of *Spiroplasma* and syn3Bsw cells. A schematic is illustrated in the left. The cell is fixed to the glass through the light gray part, and the blue part rotates. Consecutive video frames are shown for every 1/30 s. A rotational behavior of the free part is marked by blue arrows. A rotational end is marked by a red circle. (**D**) Distribution of cell helicity parameters measured by optical microscopy and EM. The *P* values by Student’s *t* test between *Spiroplasma* and syn3Bsw were 0.008, 0.002, 0.59, and 0.28 for pitch and width in optical microscopy and EM, respectively. (**E**) Consecutive video frames of swimming cells for every 0.1 s. (**F**) Change in helicity analyzed for videos shown in (E). The cell images were straightened and analyzed by ImageJ and then colored for their handedness. Helix positions traveling smoothly along the axis are traced by a yellow arrow.

**Fig. 2.**
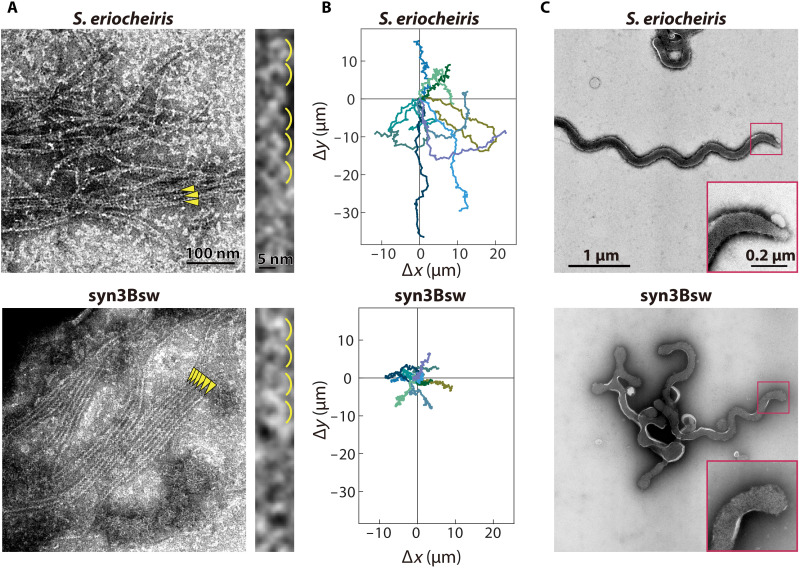
Comparison between *S. eriocheiris* and syn3Bsw. (**A**) Negative-staining EM images of filaments recovered from *Spiroplasma* and syn3Bsw cells. Filaments are marked by yellow triangles. Magnified images are illustrated in the right with marks for ring structures, characteristic for fibril filament. (**B**) Traces of a pole of 10 cells for 10 s colored differently. (**C**) Cell images under negative-staining EM images of *Spiroplasma* and syn3Bsw cells. A cell pole is magnified as inset.

We focused on *Spiroplasma eriocheiris*, an actively swimming pathogen in crustaceans (*14*). Seven genes that are likely related to swimming are encoded in four loci in the genome: *fibril*, five classes of *SmreB*, and a nonannotated conserved gene (*4*, *13*, *16*, *17*). We assembled these genes into an 8.4-kb DNA fragment and incorporated it into the syn3B genome using the Cre*/loxP* system (Fig. 1A, and fig. S1, and table S1) (*20*, *21*). An active promoter in syn3B, Ptuf, was inserted upstream of the gene cluster. Unexpectedly, under optical microscopy, 48% of the syn3B cells exhibited morphological change and active movements, presumably accompanied by force generation, and 13% had a helical shape and swimming motility (Fig. 1B and movie S1). We named this construct syn3Bsw. If we focus on cells that are partially bound to the glass, then we can observe that a free part of the cell was rotating with some reversals (Fig. 1C and movie S2), meaning that helicity switching causes helix rotation in syn3Bsw, similar to *Spiroplasma* swimming. The width and pitch of the cell helices analyzed by optical and electron microscopy (EM) were slightly different from those of *Spiroplasma* cells (Fig. 1D). Next, we analyzed the helices and handedness of cell images in each frame of the swimming video (Fig. 1, E and F). The handedness of the cell helix differed depending on its axial position, and the helicity changed over time. Furthermore, we measured the movement and rotation speed of the helix from the part where it appeared to move along the cell axis smoothly. The helix movement and rotation speeds were 8.2 ± 3.7 μm/s and 11.6 ± 4.8/s (*n* = 10), respectively, for syn3Bsw, not significantly different from 8.8 ± 2.8 μm/s and 12.0 ± 3.6/s (*n* = 16), respectively, for *Spiroplasma*. In the cryo-EM image of syn3Bsw cells, filaments running along the axis were observed in the inner part of the curvature, similar to *Spiroplasma* cells (fig. S2). The filaments recovered from syn3Bsw cells exhibited chained ring structures characteristic of the fibril filament from *Spiroplasma* cells (Fig. 2A and fig. S3) (*10*, *11*, *14*). The periodicities were 8.5 ± 0.9 nm (*n* = 41) and 8.7 ± 1.0 nm (*n* = 48) for *Spiroplasma* and syn3Bsw, respectively, without significant differences (*P* = 0.45 by Student’s *t* test). In addition, electrophoretic and mass spectrometric analyses of cell lysates indicated that fibrils and all SMreBs were expressed in syn3Bsw cells (fig. S4 and table S2). Real-time polymerase chain reaction (PCR) analyses confirmed transcription of all seven genes in both organisms (fig. S5). These results indicate that the expression of *Spiroplasma* proteins inside syn3Bsw cells resulted in the formation of internal filaments that reconstituted helical shape, helicity switching, and swimming.

### Differences in swimming between syn3Bsw and *Spiroplasma*

The speeds of helix movement and rotation were not significantly different between syn3Bsw and *Spiroplasma* (Fig. 1F). However, the trajectory of the cells over 10 s indicated that syn3Bsw could not travel long distances, unlike *Spiroplasma* (Fig. 2B). The reason can be seen in the time course of helicity switching, indicating little continuity in the rotation that hampers long-distance traveling (Fig. 1F). This may be caused by a lack of cooperativity in the helicity switching that generates helix rotation. EM images of syn3Bsw cells did not show the tapered pole, including an inner architecture called “dumbbell,” unlike *Spiroplasma* cells (Fig. 2C) (*14*), suggesting that the tapered pole made by unknown proteins plays a role in continuous helicity switching of the ribbon. Protein profile and PCR results showed that fibril protein is less abundant relative to SMreBs in syn3Bsw than in *Spiroplasma* (figs. S3 to S5). The less continuity of syn3Bsw may be related also to the smaller molar ratio of fibril protein.

### Role of component proteins

**Fig. 3.**
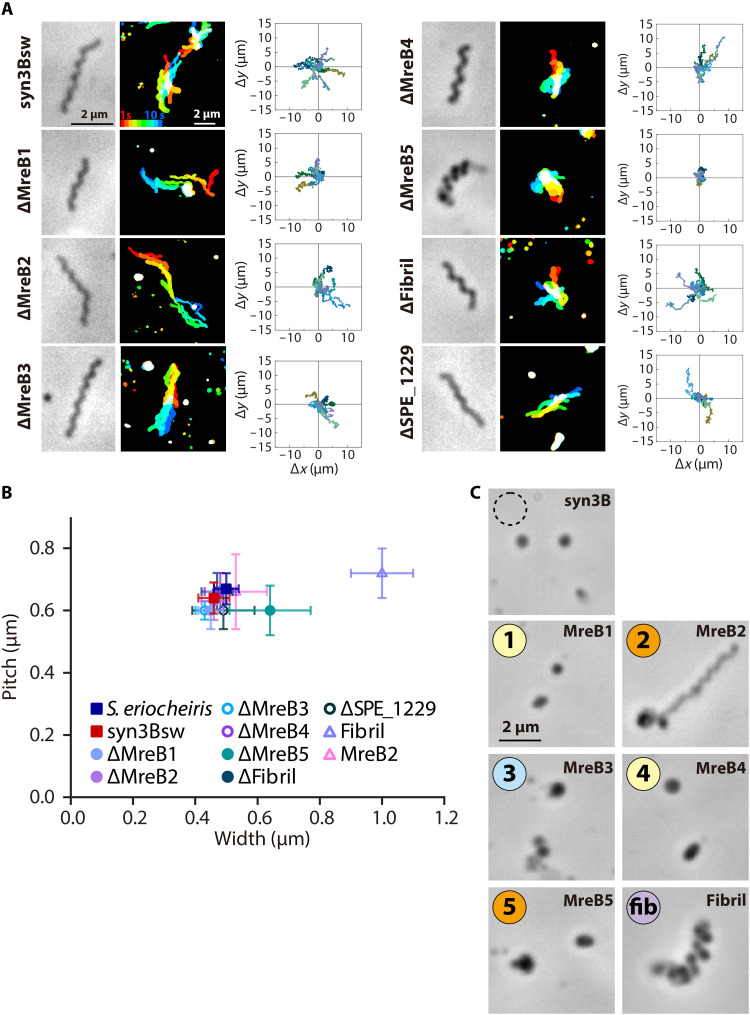
Role of individual proteins in syn3 swimming. (**A**) Structure and behaviors of cells lacking one of seven proteins from syn3sw. For each construct, phase-contrast cell image (left), integrated cell images every 1 s for 10 s with colors changing from red to blue (middle), and traces of a pole of 10 cells for 10 s (right) are indicated. (**B**) Distribution of cell helicity parameters for individual constructs analyzed with optical microscopy. (**C**) Phase-contrast image of cells expressing single *Spiroplasma* protein marked by “fib” and number of SMreBs. The original syn3B is marked by a broken circle.

To examine the role of each protein, we produced and analyzed constructs in which each protein was not expressed (Fig. 3, A and B, and movie S3). To avoid affecting gene expression by the alteration in the DNA and RNA structures, we introduced nonsense mutations to the 8th to 22nd codons of each structural gene (fig. S1). We confirmed by electrophoresis that the target proteins were no longer expressed in the mutant cells (fig. S6). No notable differences from syn3Bsw were observed in cell structures and behaviors for five of the six constructs (Fig. 3A). However, in the construct missing SMreB5, the helix width was 0.64 ± 0.13 μm, significantly larger than that of syn3Bsw in half of the filamentous cells, and the cells moved but did not swim. The distinctive features of the lack of SMreB5 are consistent with a previous observation that *Spiroplasma citri* lost helicity and swimming because of the absence of SMreB5 (*13*). These results suggest that the seven proteins have some redundant roles in helix formation and swimming.

We then examined syn3B constructs expressing each protein alone (Fig. 3C and movie S4). The cells expressing only fibril protein formed a helical cell shape with a pitch of 0.72 ± 0.08 μm and a width of 1.0 ± 0.10 μm, which is wider than *Spiroplasma* cells. The pitch of the helix is in good agreement with the number of isolated fibrils, which is consistent with the fact that fibrils are a major component of the ribbon (*10*–*12*, *14*). The cells expressing SMreB2 formed filamentous morphology, and some of them formed helices with a variety of pitches of 0.66 ± 0.12 μm. The cells expressing only SMreB1, SMreB3, SMreB4, or SMreB5 did not show differences in cell shape from the original syn3B.

### Expressing a pair of SMreBs

**Fig. 4.**
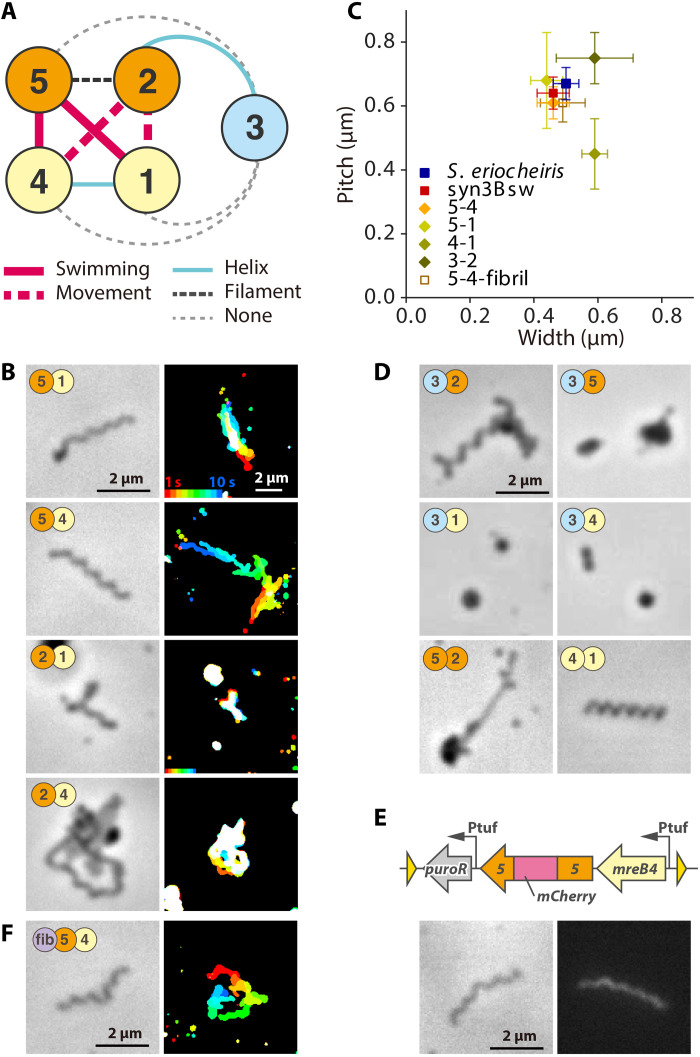
Morphology and behaviors of syn3B cells expressing pair of SMreB proteins. (**A**) Schematic of SMreB combinations with protein groups. Each SMreB is presented by a numbered circle with a group color. The characters that result in syn3B cells by gene expression are presented by line formats as follows: red solid, filamentous and helical cells with swimming motility; red broken, filamentous and helical cells with only movements; right blue solid, filamentous and helical cell without movements; black solid, filamentous cell without helicity without movements. (**B**) Image (left) and behaviors (right) of syn3B cells expressing pair of SMreBs. Cells of four constructs presented here showed movements. (**C**) Distribution of parameters for cell helicity. (**D**) Phase-contrast image of cells expressing other combinations of protein pairs. Six pairs did not show movements. (**E**) SMreB5 localization in cell expressing SMreB4 and SMreB5. Schematic of integrated genes is illustrated (top). *mCherry* gene is inserted into the C-terminal side of tyrosine residue at the 218th position. Phase-contrast and fluorescence images are illustrated (bottom). (**F**) Image (left) and behaviors (right) of syn3B cells expressing SMreB4, SMreB5, and fibril.

Furthermore, we analyzed the shapes and behaviors of cells expressing 10 combinations of SMreB protein pairs (Fig. 4A and fig. S1). We did not include fibril and SPE_1229 proteins for this search because these proteins can be removed with keeping helicity and swimming, although no other proteins can complement their roles (Fig. 3). As five classes of SMreB can be divided into three groups based on their amino acid sequence: 5-2, 4-1, and 3 (*13*, *16*, *17*), we will discuss the results based on this classification. In the pairs of SMreBs selected from the 5-2 and 4-1 groups, unexpectedly, the cells of the 5-1 and 5-4 combinations exhibited helix formation and movements, and some cells showed swimming similar to syn3Bsw, with occurrence frequencies comparable to syn3Bsw (Fig. 4, B and C, and movie S5). Cells of 2-1 showed a filamentous morphology and movements. The cells of the 2-4 combination showed filamentous morphology but were immotile; however, a few in several hundred cells showed movement. In the combinations of one of the 5-2 or 4-1 groups paired with 3, cells of 3-2 formed a right-handed helix (Fig. 4D and movie S6). In the combinations of 3-1, 3-4, and 3-5, the cells did not show differences from the original syn3B. In the combinations of the same group, 5-2 and 4-1 cells were filamentous, and 4-1 cells rarely formed a short, right-handed helix.

In the construct of 5-4, we fused the fluorescent protein mCherry into SMreB5 and SMreB4 at a position suggested by previous studies (Fig. 4E, and fig. S1, and movie S7) (*22*). The cells expressing SMreB5 fused with mCherry showed a helical cell shape and swimming, as observed in the 5-4 cells. Fluorescence was observed throughout the cell, suggesting that SMreB5 filaments were formed, although further studies are necessary for conclusion. In addition, this result indicated that mCherry fusion did not interfere with the functions of SMreB5. The 5-4 cells with mCherry fusion to SMreB4 did not exhibit conspicuous helicity. Even helical cells found in hundreds of cells did not show any movement. To clarify the roles of fibril, a major component of the ribbon structure, we analyzed cells expressing fibrils in addition to SMreB4 and SMreB5 (Fig. 4F and movie S8). The differences between the presence and absence of fibril protein were subtle in the analyses conducted in this study.

## DISCUSSION

**Fig. 5.**
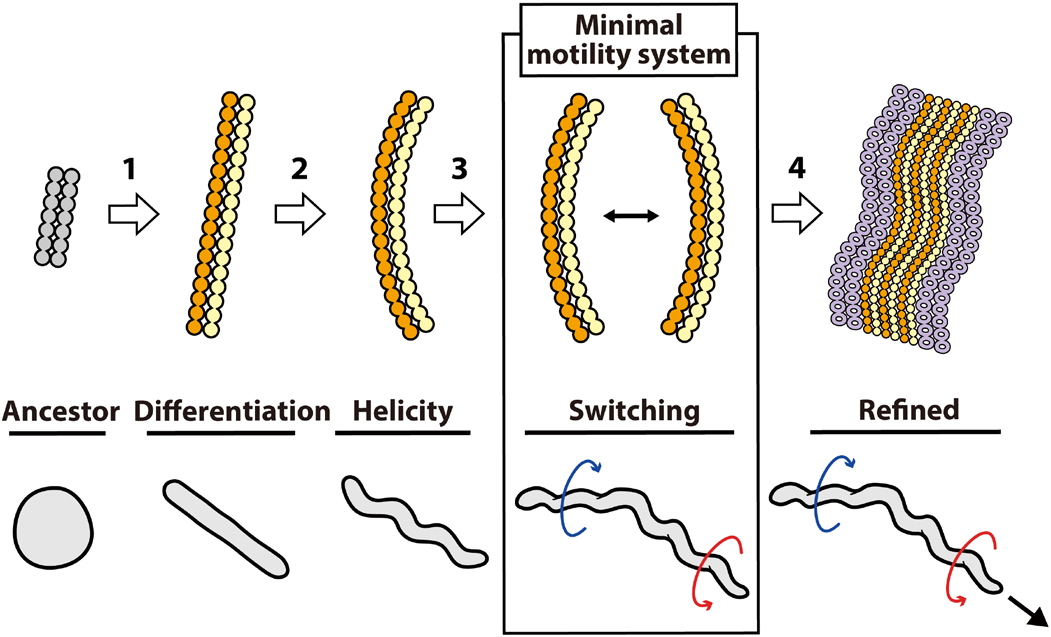
Development and mechanism of *Spiroplasma* swimming. The swimming mechanism may be acquired through four steps as denoted by white arrows. Step 1: The MreB protein derived from walled bacteria differentiated into two classes with different characters by accumulated mutations. Association of heterogeneous protofilaments allowed stable filament formation. Step 2: Small differences of protofilaments in length generated curvature, resulting in helicity of the heterogenous filament. Step 3: Change in protofilament length caused by ATP energy induces change in curvature, causing helicity switching. Then, the initial stage, which is the minimal motility system, was acquired. Step 4: The acquired swimming was refined to be equipped by five classes of SMreBs, fibril, dumbbell structure, etc. Corresponding cell morphology and behaviors are presented (bottom).

MreB belongs to the actin superfamily and forms a short antiparallel double-strand filament based on ATP energy (*23*, *24*). It has the ability to sense the curvature of the peripheral structures and serves to guide the bacterial peptidoglycan synthase to positions required for the synthesis (*25*). Isolated SMreBs also form fibers similar to those of MreB (*13*, *26*). Our results indicate that helix formation and force generation of *Spiroplasma* occur by the interaction between different SMreBs. The mechanism can be explained as follows (Fig. 5): Protofilaments made of proteins belonging to either the SMreB5-SMreB2 or SMreB4-SMreB1 groups are aligned along the cell axis and bound together. If the unit length in each protofilament is different, then some curvature is induced in the double strand, resulting in helix formation. If these protofilaments undergo a local length change at different times using ATP energy, then the curvature changes similar to a bimetallic strip, resulting in helicity switching (*4*). The length change may be related to polymerization and depolymerization in terms of the change in axial distance between the subunits. The differences in the amino acid sequence between SMreB5-SMreB2 and SMreB4-SMreB1 groups in *S. eriocheiris* range from 29.1 to 31.6% if similar amino acid pairs are excluded (*16*). These small differences suggest that the ancestors of SMreB may have acquired stability, helicity, and switching after the accidental acquisition of different properties. Specifically, it may represent the moment when a slight structural change in a housekeeping protein is amplified by an accidental accumulation of mutations, leading to motility. The reason for the existence of as many as five SMreBs, although two proteins are capable of acquiring helicity and force generation, is unclear. It may be advantageous for efficient and robust swimming, possibly in different environments, or for chemotaxis. The participation of fibril protein can be explained in a similar manner, although it forms the ribbon as the major component (*4*, *10*–*12*, *14*, *15*). This assumption is supported by our results (Fig. 2) and the facts that some swimming *Spiroplasma* species do not have fibril protein (*27*). To the best of our knowledge, the motility system comprising only two actin superfamily proteins is the smallest system established until date (*1*). Therefore, we may call this a “minimal motile cell.”

Here, we used JCVI-syn3B as the experimental platform (*18*, *19*). The lack of peptidoglycan layer in JCVI-syn3B might be advantageous for analyzing the functions of SMreB proteins because of the flexibility of cells (*28*, *29*). Because the genes of synthetic bacteria are derived from organisms related to *Spiroplasma*, it remains possible that factors derived from this near-minimal synthetic bacterium, such as proteins related to cell division (*19*) or the composition and physical properties of the cell membrane, are essential for helix formation and swimming. Thus, completely controlling factors linked to cell functions is still a future challenge. Nevertheless, the results of this study demonstrate that syn3B is a good system for studying cell functions and their evolution.

## MATERIALS AND METHODS

### Bacterial strains and culture conditions

JCVI-syn3B (GenBank, CP069345.1), *S. eriocheiris* (TDA-040725-5 T), and *Escherichia coli* (DH5α) for DNA manipulation were cultured in SP4 (*18*, *19*), SP4, and LB media at 37°, 30°, and 37°C, respectively.

### Plasmid construction

The *Spiroplasma* genome was isolated as previously described (*30*). The plasmid used to transform JCVI-syn3B to obtain syn3Bsw (pSeW001) was constructed as follows (fig. S1). Focused *Spiroplasma* DNA regions, *puroR* gene, and vector fragment were amplified from the *Spiroplasma* genome DNA and pSD079 DNA (*21*) as five PCR products, using the primer sets listed in table S1. The DNA fragments were assembled using the In-Fusion HD Cloning Kit (Takara Bio Inc., Kusatsu, Japan). pSeW002 was constructed by replacing the upstream region of the first gene, a *fibril* in pSeW001 with a Ptuf fragment [promoter from the EF-Tu (Elongation Factor Thermo Unstable) gene] amplified from pSD079. pSeW102, pSeW202, pSeW302, pSeW402, pSeW502, pSeW602, and pSeW702 were modified to introduce nonsense mutations in individual genes. The plasmids used to construct other strains were modified from pSeW005, which was constructed by the process described above, using pSeW002 as the PCR template. Ptuf or Pspi (Spiralin promoter from *Spiroplasma*) (*21*) were inserted at the 5′ end of the open reading frame. All the DNA fragments were verified for DNA sequences.

### Transformation and cell preparation

The transformation of JCVI-syn3B was performed as previously described (*31*) with two modifications: (i) The entire process was scaled down by a factor of 15, and (ii) the mixture of cells and DNA was kept on ice for 10 min to increase its transformation efficiency. The transformant colonies were picked and inoculated into 200 μl of SP4 medium containing puromycin (3 μg/ml), cultured for 18 to 24 hours, and confirmed for transformation by PCR. The cells were cultured in a liquid medium a few more times, with an inoculation of 100 to 500 dilution, and then frozen as stock. To analyze the cells, the frozen stock was inoculated at 100 to 500 dilution in SP4 medium with puromycin and grown for 20 to 24 hours at 37°C. Cultures at an optical density at 620 nm of 0.03 were used for the analyses of JCVI-syn3B and *S. eriocheiris*.

### Optical microscopy and protein profiling

The cultured cells of syn3B and *Spiroplasma* were analyzed in a 0.5× SP4 medium diluted with phosphate-buffered saline (PBS), containing 0.5% methylcellulose and bovine serum albumin (0.5 mg/ml). If necessary, the cell density was adjusted by centrifugation at 11,000*g* at 10°C for 10 min, followed by suspension with the diluted medium. The cell suspension was inserted into a tunnel slide (*14*, *30*, *32*, *33*) and observed using an inverted microscope IX71 (Olympus, Tokyo, Japan) equipped with a UPlanSApo 100× 1.4 numerical aperture Ph3 and complementary metal-oxide semiconductor (CMOS) camera, DMK33UX174 (The Imaging Source Asia Co. Ltd. Taipei, Taiwan). The videos were analyzed by ImageJ ver.1.53f51 (Fiji) using plugins, MTrackJ, empirical gradient threshold, and a color footprinting macro (*34*). Profiling and identification of proteins in cells were performed as previously described (*14*, *35*, *36*).

### Electron microscopy

To observe the intact cells, cultured cells were collected by centrifugation, suspended to a 10-fold density of the original in the medium, and fixed using 0.5% glutaraldehyde for 5 min at 25°C. After quenching with 500 mM tris-HCl (pH 7.5), the cells were collected by centrifugation, washed, and suspended in PBS to a 40-fold density of the original. The cell suspension was placed on a carbon-coated grid for 5 min, removed, rinsed with PBS thrice, and then stained with 2% phosphotungstic acid for 60 s. To observe the internal structure, the cell suspension was treated with PBS containing 1% Triton X-100, deoxyribonuclease (0.1 mg/ml), 1 mM MgCl_2_, and 1 mM phenylmethylsulfonyl fluoride for 10 min at 4°C, and centrifuged at 20,000*g* for 30 min at 4°C. The pellet was suspended in PBS to a 160-fold density of the original, placed on the EM grid for 2 min, and stained with 2% phosphotungstic acid for 60 s. Images were acquired using a JEM1010 EM (JEOL, Akishima, Japan) equipped with a FastScan-F214(T) charge-coupled device camera (TVIPS, Gauting, Germany). For cryo-EM, the cultured cells were collected, suspended to a 10-fold density of the original, and frozen as described previously (*37*). The images were captured using a Talos F200C EM (Thermo Fisher Scientific, Waltham, MA, USA) equipped with a 4000 × 4000 Ceta CMOS camera (Thermo Fisher Scientific). The images were analyzed using ImageJ software.

### Real-time PCR

RNA was extracted using RNeasy mini kit (QIAGEN, Venlo, the Netherlands). Reverse transcription was performed using ReverTra Ace (Toyobo, Osaka, Japan). Real-time PCR was performed using PCR SsoAdvanced Universal SYBR Green Supermix (Bio-Rad Laboratories, Hercules, USA) in Thermal cycler CFX cennect (Bio-Rad). Primers were designed by Primer blast (*38*). Absolute quantification was performed using pSeW002 as the standard.

## Supplementary Materials

### This PDF file includes:

Figs. S1 to S6 Tables S1 and S2 References
