# Supplementary material for: Reconstitution of a minimal motility system based on Spiroplasma swimming by two bacterial actins in a synthetic minimal bacterium
Source: Sci Adv. 2022 Nov 30;8(48):eabo7490. doi: 10.1126/sciadv.abo7490 (PMC9710875; doi:10.1126/sciadv.abo7490)
Supplement: Supplementary file 1 — Figs. S1 to S6 Tables S1 and S2 References [file sciadv.abo7490_sm.pdf]

Science Advances
AAAS

Supplementary Materials for

Reconstitution of a minimal motility system based on *Spiroplasma* swimming by two bacterial actins in a synthetic minimal bacterium

Hana Kiyama *et al.*

Corresponding author: Makoto Miyata, miyata@omu.ac.jp

Sci. Adv. **8**, eabo7490 (2022)
DOI: 10.1126/sciadv.abo7490

The PDF file includes:

Figs. S1 to S6
Tables S1 and S2
Legends for movies S1 to S8
Legend for data file S1
References

Other Supplementary Material for this manuscript includes the following:

Movies S1 to S8
Data file S1

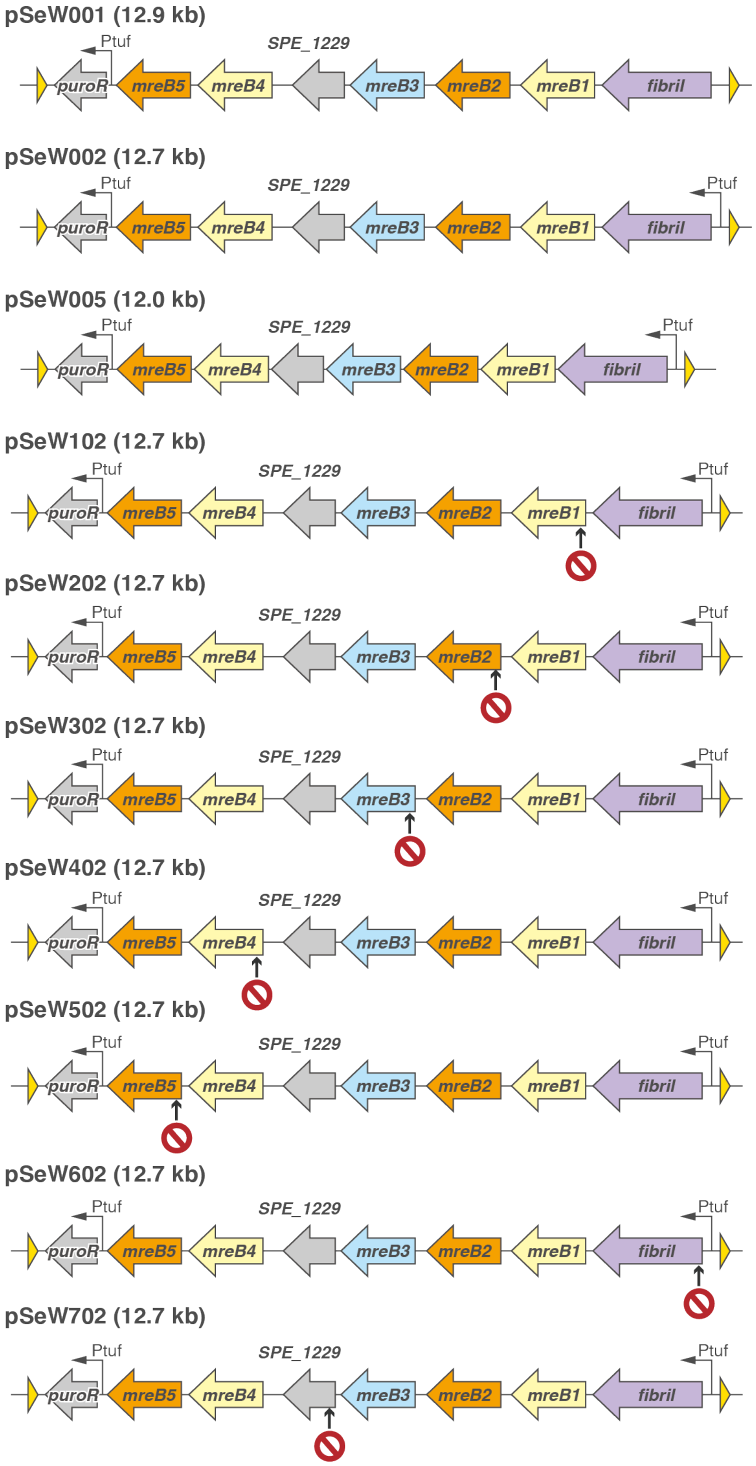
pSeW001 (12.9 kb)
Ptuf
SPE_1229
puroR mreB5 mreB4 mreB3 mreB2 mreB1 fibril
pSeW002 (12.7 kb)
Ptuf
SPE_1229
Ptuf
puroR mreB5 mreB4 mreB3 mreB2 mreB1 fibril
pSeW005 (12.0 kb)
Ptuf
SPE_1229
Ptuf
puroR mreB5 mreB4 mreB3 mreB2 mreB1 fibril
pSeW102 (12.7 kb)
Ptuf
SPE_1229
Ptuf
puroR mreB5 mreB4 mreB3 mreB2 mreB1 fibril
pSeW202 (12.7 kb)
Ptuf
SPE_1229
Ptuf
puroR mreB5 mreB4 mreB3 mreB2 mreB1 fibril
pSeW302 (12.7 kb)
Ptuf
SPE_1229
Ptuf
puroR mreB5 mreB4 mreB3 mreB2 mreB1 fibril
pSeW402 (12.7 kb)
Ptuf
SPE_1229
Ptuf
puroR mreB5 mreB4 mreB3 mreB2 mreB1 fibril
pSeW502 (12.7 kb)
Ptuf
SPE_1229
Ptuf
puroR mreB5 mreB4 mreB3 mreB2 mreB1 fibril
pSeW602 (12.7 kb)
Ptuf
SPE_1229
Ptuf
puroR mreB5 mreB4 mreB3 mreB2 mreB1 fibril
pSeW702 (12.7 kb)
Ptuf
SPE_1229
Ptuf
puroR mreB5 mreB4 mreB3 mreB2 mreB1 fibril

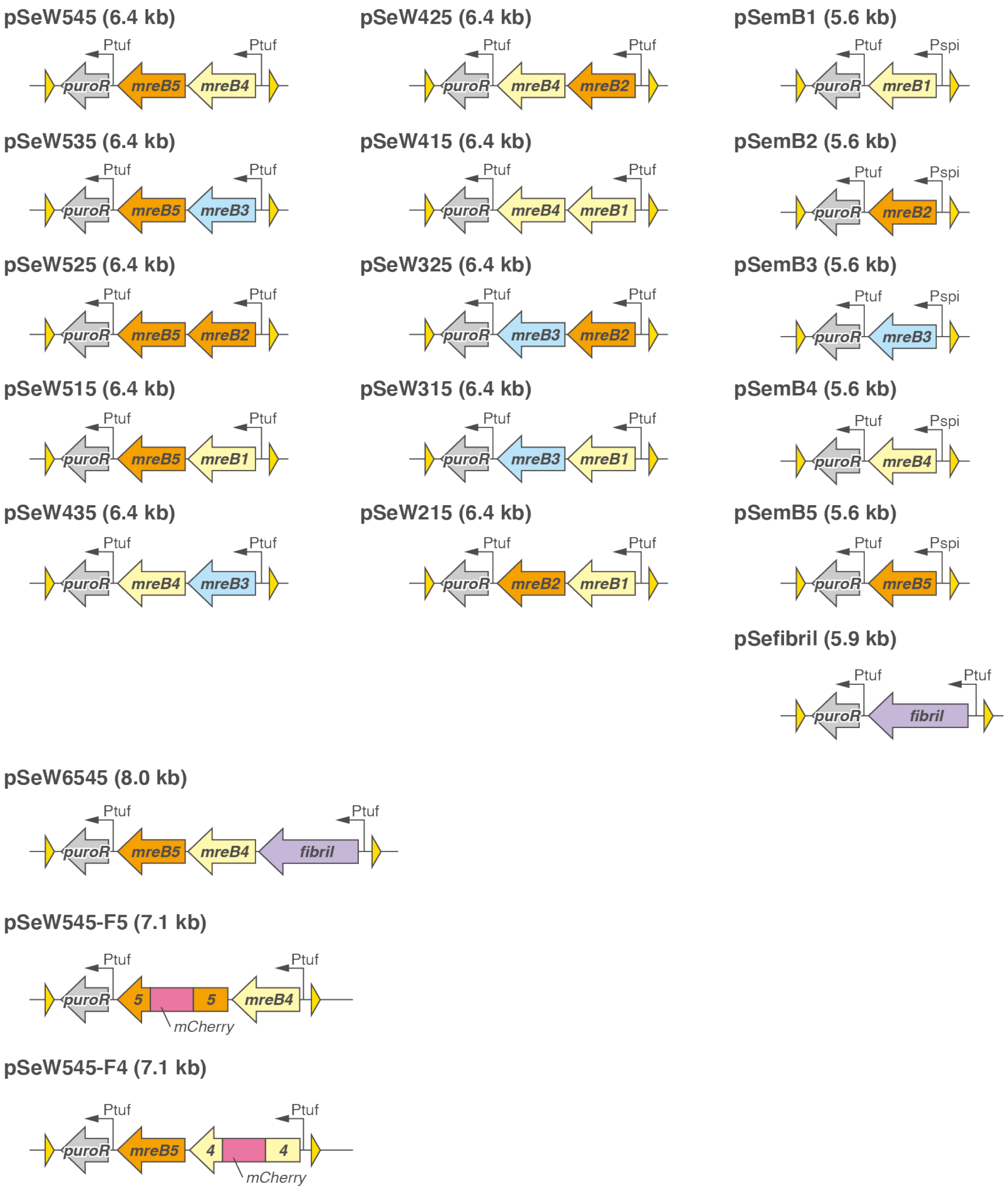

Fig. S1.

Schematic presentation for DNA constructs used in this study.

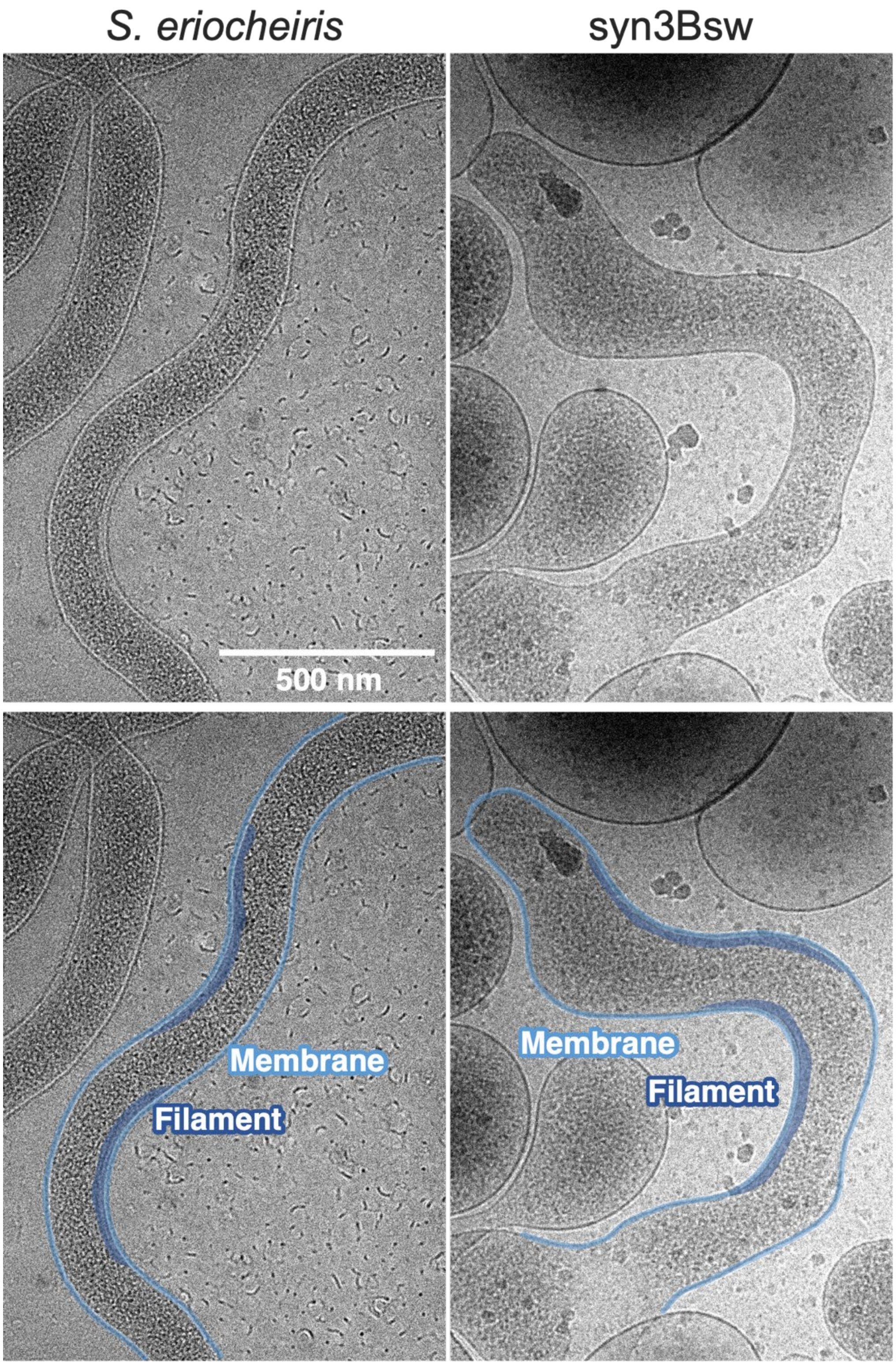

Fig. S2.
Cell images under cryo electron microscopy. In the lower panels, the cell membrane and filamentous structures are colored light and dark blue, respectively.

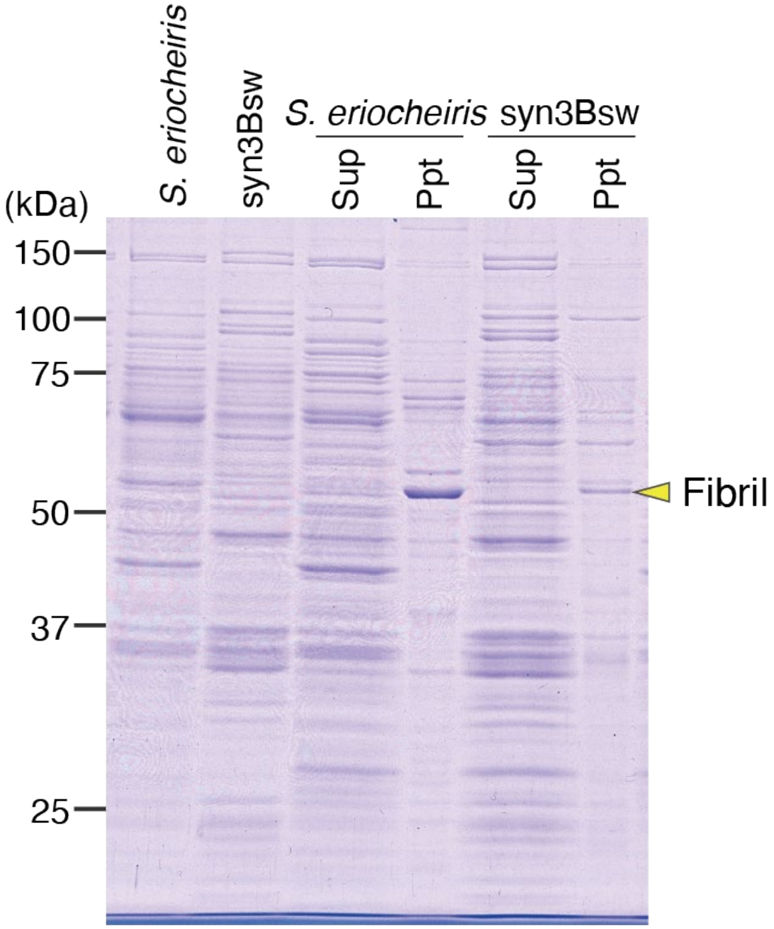

Fig. S3.

Identification of fibril protein in syn3Bsw. The cultured cells were washed, lyzed , fractionated by centrifugation, and analyzed by SDS-10% PAGE. The entire cell lysates are illustrated in the left two lanes. Fibril protein is marked. Protein bands were stained by Coomassie Brilliant Blue.

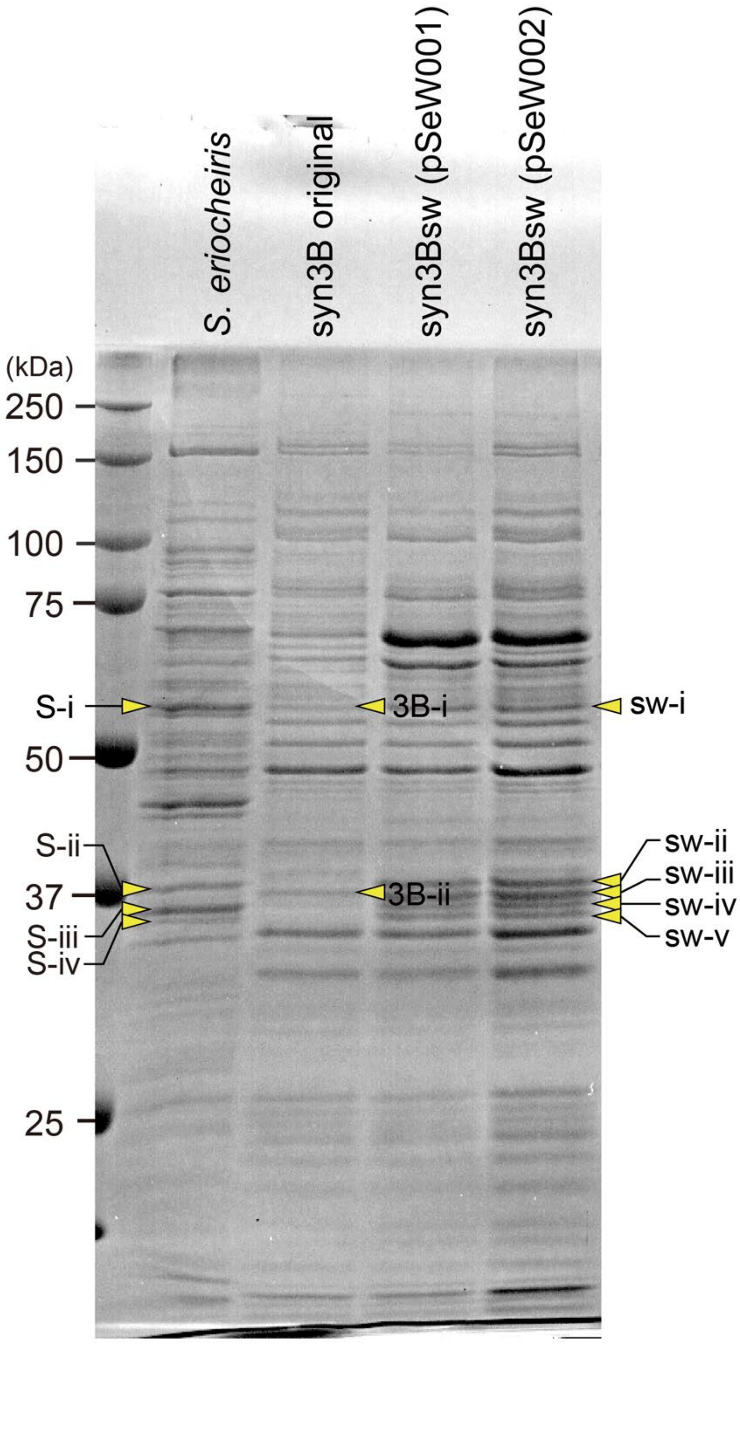

Fig. S4.

Protein profiles of cell lysates visualized by SDS-12.5% PAGE stained by Coomassie Brilliant Blue. Constructs are indicated on the top, and focused protein bands marked by a yellow triangle with a number were identified by PMF as shown in Table S2.

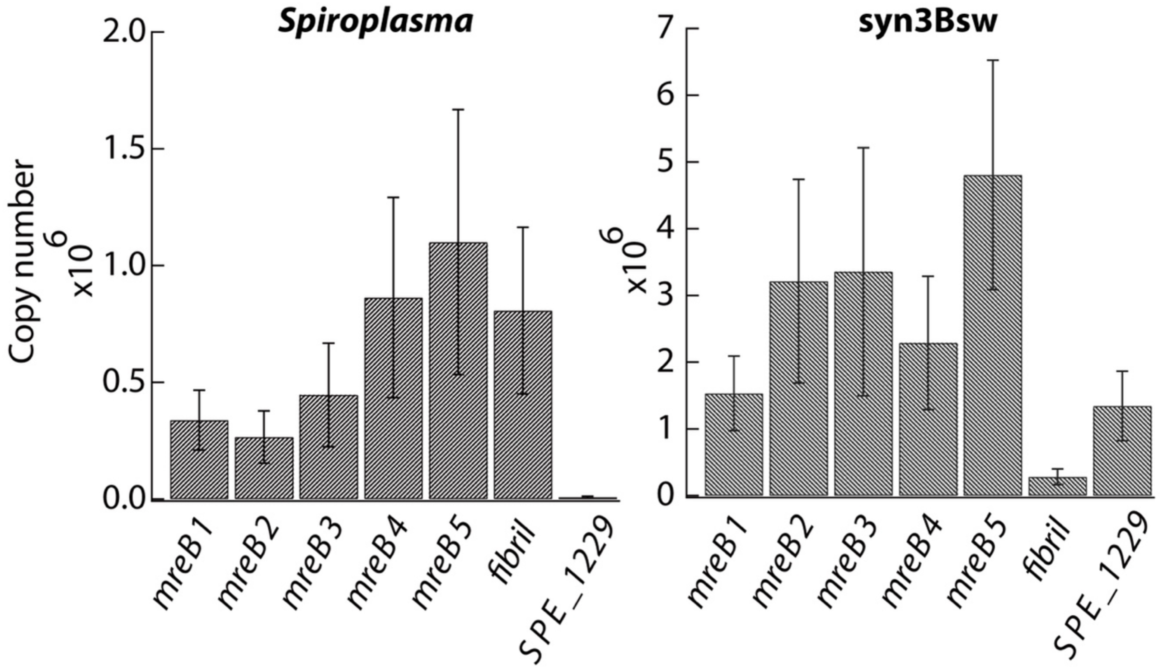

Fig. S5.
Transcription levels of the seven genes in *Spiroplasma* and syn3Bsw. The copy numbers in 0.67 μL culture were estimated by real-time PCR. pSeW002 plasmid was used as the standard.

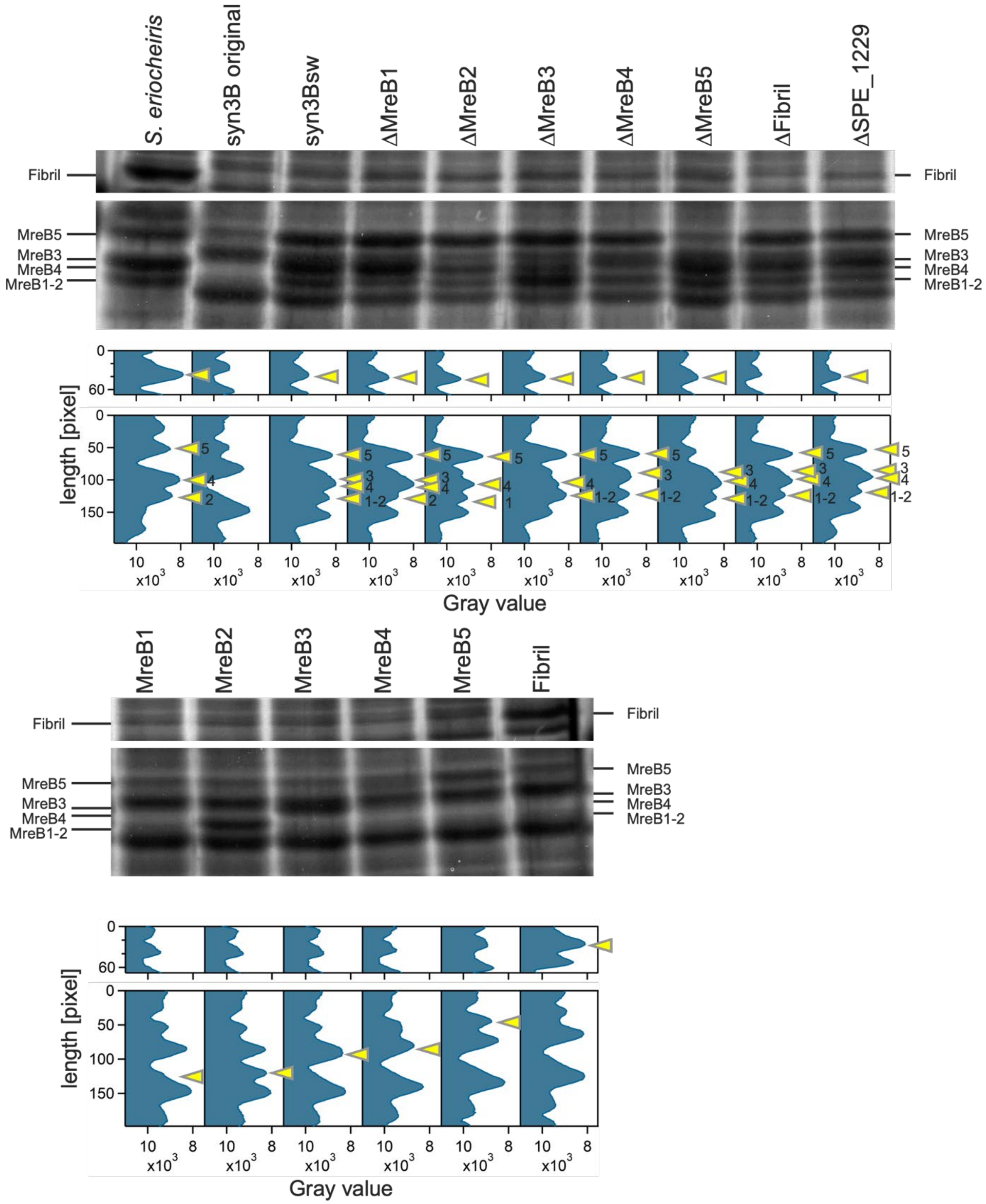
S. eriocheiris
syn3B original
syn3Bsw
ΔMreB1
ΔMreB2
ΔMreB3
ΔMreB4
ΔMreB5
ΔFibril
ΔSPE_1229
Fibril
MreB5
MreB3
MreB4
MreB1-2
length [pixel]
Gray value
x10^3
MreB1
MreB2
MreB3
MreB4
MreB5
Fibril

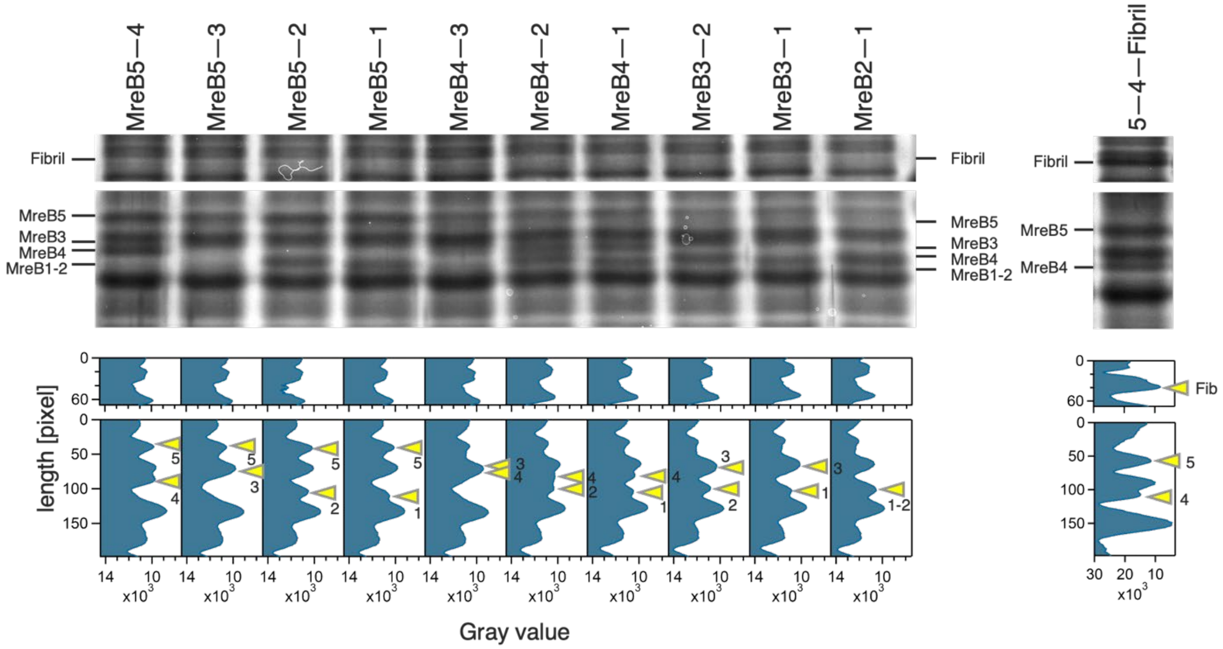

Fig. S6.

Focused proteins visualized by SDS-10% PAGE stained by reverse staining method. Protein bands (upper) were analyzed for the band intensity (lower). Constructs are indicated on the top. Proteins are indicated on the both sides of upper panel and by yellow triangles on the lower panel.

Table S1.

DNA primers used in this study.

Name	Sequence	Purpose
w_mreb3to5_R	TATAAAATTTAAAAATTACTTGGTATTGATAGTAAG	pSeW001
w_mreb2_F	ACCAAGTAATTTTTAAATTTTATATTCTTTTGCTTC	pSeW001
w_mreb2_R	TTAGATTATTAAATTCTCCCTCACTAAAACTAGTTG	pSeW001
w_mreb1_F	GTGAGGGAGAATTTAATAATCTAATTCTTTGTTTTT	pSeW001
w_mreb1_R	AAAAGTGAATAATTGCCTGAAGATTTAATAATAATT	pSeW001
w_fibril_F	ATCTTCAGGCAATTATTCACTTTTTAAACGAATAGT	pSeW001
w_fibril_R	TTGACAGCTAGCTCAGTCCTACCTTAAGTTTACTGGATTTTTAAG	pSeW001
LP_inverse_R	GAACTATATAAATAACTCGCATATTG	pSeW001, pSemB1, pSemB2, pSemB3, pSemB4, pSemB5, pSefibril, pSeW435, pSeW425, pSeW415, pSeW325, pSeW315, pSeW215
LP_inverse_F	AGGACTGAGCTAGCTGTCAAAGATC	pSeW001, pSemB2, pSefibril
w_mreb3to5_F	GCGAGTTATTTATATAGTTCTTATTTTTTCTTCCCAATGCCAGC	pSeW001, pSemB5
addFib-to079-F	GAGTTATTTATATAGTTCTTATTCACTTTTTAAACGAATAGT	pSeW002, pSefibril
addFib-toPtuf-R	TTTTAAGGAGAAAAAAACATGATTGGAGTTATTTCAACTGCG	pSeW002, pSefibril
Ptuf-to079-R	GACAGCTAGCTCAGTCCTTATTTTTTGAATTAAGTATTAAAT	pSeW002, pSefibril
Ptuf-F	GTTTTTTTCTCCTTAAAATTTCTATAAC	pSeW002, pSefibril, pSeW545, pSeW535, pSeW525, pSeW515, pSeW435, pSeW425, pSeW415, pSeW325, pSeW315, pSeW215
Stop-mreB1-F	TACAAAAGTCGGTTACTTATTGTTAATC	pSeW102
Stop-mreB1-R	CAATAAGTAACCGACTTTTGTATCAATT	pSeW102
Stop-mreB2-F	TACGCGACAGTTTATGCAGTTCCTAA	pSeW202
Stop-mreB2-R	GAACTGCATAAACTGTCGCGTATGTC	pSeW202
Stop-mreB3-F	ACGAGGTGGTTAAGGAGAAATATTAAATG	pSeW302
Stop-mreB3-R	TTTCTCCTTAACCACCTCGTAAATTTATTG	pSeW302
stop-mreB4-F	GAAAGTTGGTTATTTACTTTTGCCGCTA	pSeW402
stop-mreB4-R	AAAGTAAATAACCAACTTTCGTTTCAAT	pSeW402
Stop-mreB5-F2	CCCTTGACCTTAAACGTAAGCTAACACG	pSeW502
Stop-mreB5-R2	CTTACGTTTAAGGTCAAGGGATTATCTA	pSeW502
MreB1-F	GCGAGTTATTTATATAGTTCTTAATAATCTAATTCTTTGTTTTT	pSemB1
MreB1-Pspi-R	GAGAAAGGAAATATAAGATCATGGCATTGATTAACAATAAGAAA	pSemB1
Pspi_inverse-F	GATCTTATATTTCCTTTCTCTATT	pSemB1, pSemB3, pSemB4, pSemB5
MreB2_R	atggctaattataaatttggaaaa	pSemB2
MreB2_Pspi_F	aaatttataattagccatGATCTTATATTTCCTTTCTCTATTAAGTAG	pSemB2
LP_insert_Pspi_R	GACAGCTAGCTCAGTCCTAATTAAAAGTTAGTGAACAAGAAA	pSemB2
LP_insert_MreB2_F	GAGTTATTTATATAGTTCTTAAATTTTATATTCTTTTGCTTC	pSemB2, pSeW215
Mreb3-Pspi-R	GAGAAAGGAAATATAAGATCATGACTATAACAGACGTATTAAAA	pSemB3
MreB3-F	GCGAGTTATTTATATAGTTCTTATTTATTTTTTTTATTTTCTTC	pSemB3, pSeW325, pSeW315
MreB4-Pspi-R	GAGAAAGGAAATATAAGATCATGTTAGATATTGTTTATGTTTAT	pSemB4
MreB4-F	GCGAGTTATTTATATAGTTCTTAGTAATCTAATTCTTTAGTATG	pSemB4, pSeW435, pSeW425, pSeW415
MreB5-Pspi-R	GAGAAAGGAAATATAAGATCGTGAAACCAGAAAGACCATTTATC	pSemB5
mreB4-Ptuf-ORF-R	AATTTTAAGGAGAAAAAAACATGGCAGGATTTAATAGCGGCAA	pSeW545
SD-mreB5-R	ATATTAAGGAGGAAATTAACGTGAA	pSeW535, pSeW525, pSeW515
mreB3-Ptuf-R	AATTTTAAGGAGAAAAAAACATGACTATAACAGACGTATTAAAA	pSeW535, pSeW435

mreB3-SD-mreB5-F	GTTAATTTCCTCCTTAATATTTATTTATTTTTTTTATTTTCTTCAATT	pSeW535
mreB2- Ptuf-R	AATTTTAAGGAGAAAAAAACATGGCTAATTATAAATTTGGAAAA	pSeW525, pSeW425, pSeW325
mreB2- SD-mreB5-F	GTTAATTTCCTCCTTAATATTTAAATTTTATATTCTTTTGCTTC	pSeW525
mreB1- Ptuf-R	AATTTTAAGGAGAAAAAAACATGGCATTGATTAACAATAAGAAA	pSeW515, pSeW415, pSeW315, pSeW215
mreB1- SD-mreB5-F	GTTAATTTCCTCCTTAATATTTAATAATCTAATTCTTTGTTTTTG	pSeW515
mreB3-mreB4-F	CCTCCCTTTTTTATTTATTTTTTTTATTTTCTTC	pSeW435
mreB4-mreB3-R	AAATAAATAAAAAAGGGAGGAATTTTTACAATG	pSeW435
mreB2_mreB4_F	CCTCCCTTTTTTAAATTTTATATTCTTTTGCTTC	pSeW425
mreB2_mreB4_R	TAAAATTTAAAAAAGGGAGGAATTTTTACAAT	pSeW425
mreB1-only-F	TTAATAATCTAATTCTTTGTTTTTGA	pSeW415, pSeW315
mreB4-mreB1-R	ACAAAGAATTAGATTATTAAAAAAGGGAGGAATTTTTACAATG	pSeW415
mreB3-mreB1-R	ACAAAGAATTAGATTATTAAATGAAAGGAGACAATCATAGATG	pSeW315
mreB4-mChe-F	ATAATAGCTGATGATCCTGAGTATTTTGCTAATGAACCAATTTC	pSeW545-F4
mreB4-mChe-R	ATAAAAGTGGAGCTCCTGGTCCAGACGAAAGAAAAATGAAAGTT	pSeW545-F4
mreB5-mChe-F	ATAATAGCTGATGATCCTGAGTATTTAACTAATGAACCGATGTA	pSeW545-F5
mreB5-mChe-R	ATAAAAGTGGAGCTCCTGGTCATAATGAACGTGCAATGCAAATT	pSeW545-F5
SWmCh-linker-R2	TCAGGATCATCAGCTATTATTAAAGAATTT	pSeW545-F5, pSeW545-F4
SWmCh-linker-F	ACCAGGAGCTCCACTTTTATATAGTTCATCCATACCAC	pSeW545-F5, pSeW545-F4
mreB4-SD-fibril-R	TTCGTTTAAAAAGTGAATAAAAAAGGGAGGAATTTTTACAATGG	pSeW6545
fibril-F	TTATTCACTTTTTAAACGAATAGTTAC	pSeW6545
coloP-puroR-F	ggagtagtccaaacagcaacagca	colony PCR
syn3B-junc-F	TATGTGATAATGCCAATCGCTAAG	colonyPCR
syn3B-junc-R	GTAAATTCCCAAATTATTCCATCA	colonyPCR
q_spe_mreB4_F	GCAAAATACCCAGACGAAAG	Real-time PCR
q_spe_mreB4_R	TCTTCTGGTGTAACTTCGAT	Real-time PCR
q_spe_mreB5_F	GAGGAACAACTGACTTAGCA	Real-time PCR
q_spe_mreB5_R	CATCATCAAAGTGGTTTCCG	Real-time PCR
q_spe_fibril_F	CTACCAAATTGTTTGTCTTC	Real-time PCR
q_spe_fibril_R	TAGTTAATTGTGATGCAAGA	Real-time PCR
60q_spe_mreB1_F	CATTGTCCGTCCAATGGTTG	Real-time PCR
60q_spe_mreB1_R	TGTTTTGAGACATGTAAACGGT	Real-time PCR
60q_spe_mreB2_F	ATTTGTGGAGGTGGTGCCTT	Real-time PCR
60q_spe_mreB2_R	ATTAGGGGATCTTGAGCGGC	Real-time PCR
60q_spe_mreB3_F	TGGAAACCGTGCCAAAGTTC	Real-time PCR
60q_spe_mreB3_R	ACCATTGCGCGACGTTCTAA	Real-time PCR
60q_spe_mreB5_F	AATGGCAGCAATTGGAGCAG	Real-time PCR
60q_spe_mreB5_R	TGCTAAGTCAGTTGTTCCTCCT	Real-time PCR
60q_spe_fibril_F	GGGGAACAGTTGCTCCGTTAT	Real-time PCR
60q_spe_fibril_R	TGGCTAACGAAATTGCTGCTG	Real-time PCR
60q_spe_ldh_F	CCCCTGCTTCGACACTAACA	Real-time PCR
60q_spe_ldh_R	GACTTGGCGGAAGGAAATGC	Real-time PCR
60q_SPE1229_F	CCCTGACGAGTAGCTTAGCA	Real-time PCR
60q_SPE1229_R	GTTCAACTAGTAAAAGCCTGTTTGA	Real-time PCR

Table S2.

Protein identification by mass spectrometry for Fig. S4.

Bacterial strain	Protein band	Gene ID	Annotation	Mass (kDa)	score*	coverage (%)
S. eriocheiris	S-i	SPE_0666	Fibril	58.7	184	63
S. eriocheiris	S-ii	SPE_1231	MreB5	38.7	117	54
S. eriocheiris	S-iii	SPE_1230	MreB4	40.7	64	49
S. eriocheiris	S-iii	SPE_1049	GAPDH	35.8	129	68
S. eriocheiris	S-iv	SPE_1224	MreB2	37.9	55	36

original syn3B	3B-i	ODP2_MYCCT	2-oxo acid dehydrogenase subunit E2	47	52	24
original syn3B	3B-ii	–	–	–	–	–
syn3Bsw	sw-i	SPE_0666	Fibril	58.7	124	52
syn3Bsw	sw-i	ODP2_MYCCT	2-oxo acid dehydrogenase subunit E2	47	69	36
syn3Bsw	sw-ii	SPE_1231	MreB5	38.7	131	57
syn3Bsw	sw-iii	SPE_1228	MreB3	38.5	187	71
syn3Bsw	sw-iv	SPE_1230	MreB4	40.7	86	57
syn3Bsw	sw-iv	SPE_1228	MreB3	38.5	89	48
syn3Bsw	sw-iv	SPE_1224	MreB2	37.9	46	34
syn3Bsw	sw-v	SPE_1224	MreB2	37.9	101	42
syn3Bsw	sw-v	SPE_0470	MreB1	38	96	45

*Score was provided by Mascot search (*39*). Protein scores greater than 47 are significant ($p<0.05$).

Movie S1.

Cell behaviors of three strains indicated on top. Real-time movie for 5 s.

Movie S2.

Rotational behaviors of freely moving site of *Spiroplasma* and syn3Bsw cells for 10 s.

Movie S3.

Cells lacking one of seven proteins from syn3sw for 10 s.

Movie S4.

Cells expressing a single *Spiroplasma* protein for 5 s.

Movie S5.

Syn3B cells expressing a pair of SMreBs for 10 s.

Movie S6.

Cells expressing a single protein for 10 s.

Movie S7.

SMreB5 localization in cell expressing SMreB 4 and 5 visualized by fluorescence for 10 s.

Movie S8.

syn3B cells expressing SMreBs 4, 5, and fibril for 10 s.

Data S1. (separate file)

DNA sequences of constructs used in this study.

REFERENCES AND NOTES

1. M. Miyata, R. C. Robinson, T. Q. P. Uyeda, Y. Fukumori, S. I. Fukushima, S. Haruta, M. Homma, K. Inaba, M. Ito, C. Kaito, K. Kato, T. Kenri, Y. Kinosita, S. Kojima, T. Minamino, H. Mori, S. Nakamura, D. Nakane, K. Nakayama, M. Nishiyama, S. Shibata, K. Shimabukuro, M. Tamakoshi, A. Taoka, Y. Tashiro, I. Tulum, H. Wada, K. I. Wakabayashi, Tree of motility - A proposed history of motility systems in the tree of life. *Genes Cells* **25**, 6–21 (2020).

2. H. Grosjean, M. Breton, P. Sirand-Pugnet, F. Tardy, F. Thiaucourt, C. Citti, A. Barré, S. Yoshizawa, D. Fourmy, V. de Crécy-Lagard, A. Blanchard, Predicting the minimal translation apparatus: Lessons from the reductive evolution of mollicutes. *PLOS Genet.* **10**, e1004363 (2014).

3. S. Razin, L. Hayflick, Highlights of mycoplasma research—An historical perspective. *Biologicals* **38**, 183–190 (2010).

4. Y. Sasajima, M. Miyata, Prospects for the mechanism of *Spiroplasma* swimming. *Front. Microbiol.* **12**, 706426 (2021).

5. M. Miyata, T. Hamaguchi, Integrated information and prospects for gliding mechanism of the pathogenic bacterium *Mycoplasma pneumoniae*. *Front. Microbiol.* **7**, 960 (2016).

6. M. Miyata, T. Hamaguchi, Prospects for the gliding mechanism of *Mycoplasma mobile*. *Curr. Opin. Microbiol.* **29**, 15–21 (2016).

7. D. Nakane, T. Ito, T. Nishizaka, Coexistence of two chiral helices produces kink translation in *Spiroplasma* swimming. *J. Bacteriol.* **202**, e00735-19 (2020).

8. H. Wada, R. R. Netz, Hydrodynamics of helical-shaped bacterial motility. *Phys. Rev. E Stat. Nonlin. Soft Matter Phys.* **80**, 021921 (2009).

9. J. W. Shaevitz, J. Y. Lee, D. A. Fletcher, *Spiroplasma* swim by a processive change in body helicity. *Cell* **122**, 941–945 (2005).

10. Y. Sasajima, T. Kato, T. Miyata, A. Kawamoto, K. Namba, M. Miyata, Isolation and structure of the fibril protein, a major component of the internal ribbon for *Spiroplasma* swimming. *Front. Microbiol.* **13,** 1004601 (2022).

11. S. Cohen-Krausz, P. C. Cabahug, S. Trachtenberg, The monomeric, tetrameric, and fibrillar organization of Fib: The dynamic building block of the bacterial linear motor of *Spiroplasma melliferum* BC3. *J. Mol. Biol.* **410**, 194–213 (2011).

12. J. Kürner, A. S. Frangakis, W. Baumeister, Cryo-electron tomography reveals the cytoskeletal structure of *Spiroplasma melliferum*. *Science* **307**, 436–438 (2005).

13. S. Harne, S. Duret, V. Pande, M. Bapat, L. Béven, P. Gayathri, MreB5 Is a determinant of rod-to-helical transition in the cell-wall-less bacterium *Spiroplasma*. *Curr. Biol.* **30**, 4753–4762.e7 (2020).

14. P. Liu, H. Zheng, Q. Meng, N. Terahara, W. Gu, S. Wang, G. Zhao, D. Nakane, W. Wang, M. Miyata, Chemotaxis without conventional two-component system, based on cell polarity and aerobic conditions in helicity-switching swimming of *Spiroplasma eriocheiris*. *Front. Microbiol.* **8**, 58 (2017).

15. S. Trachtenberg, L. M. Dorward, V. V. Speransky, H. Jaffe, S. B. Andrews, R. D. Leapman, Structure of the cytoskeleton of *Spiroplasma melliferum* BC3 and its interactions with the cell membrane. *J. Mol. Biol.* **378**, 778–789 (2008).

16. D. Takahashi, I. Fujiwara, M. Miyata, Phylogenetic origin and sequence features of MreB from the wall-less swimming bacteria *Spiroplasma*. *Biochem. Biophys. Res. Commun.* **533**, 638–644 (2020).

17. C. Ku, W. S. Lo, C. H. Kuo, Molecular evolution of the actin-like MreB protein gene family in wall-less bacteria. *Biochem. Biophys. Res. Commun.* **446**, 927–932 (2014).

18. C. A. Hutchison III, R.-Y. Chuang, V. N. Noskov, N. Assad-Garcia, T. J. Deerinck, M. H. Ellisman, J. Gill, K. Kannan, B. J. Karas, L. Ma, J. F. Pelletier, Z.-Q. Qi, R. A. Richter, E. A. Strychalski, L. Sun, Y. Suzuki, B. Tsvetanova, K. S. Wise, H. O. Smith, J. I. Glass, C.

Merryman, D. G. Gibson, J Craig Venter, Design and synthesis of a minimal bacterial genome. *Science* **351**, aad6253 (2016).

19. J. F. Pelletier, L. Sun, K. S. Wise, N. Assad-Garcia, B. J. Karas, T. J. Deerinck, M. H. Ellisman, A. Mershin, N. Gershenfeld, R.-Y. Chuang, J. I. Glass, E. A. Strychalski, Genetic requirements for cell division in a genomically minimal cell. *Cell* **184**, 2430–2440.e16 (2021).

20. F. Nishiumi, Y. Kawai, Y. Nakura, M. Yoshimura, H. N. Wu, M. Hamaguchi, S. Kakizawa, Y. Suzuki, J. I. Glass, I. Yanagihara, Blockade of endoplasmic reticulum stress-induced cell death by *Ureaplasma parvum* vacuolating factor. *Cell Microbiol.* **23**, e13392 (2021).

21. A. M. Mariscal, S. Kakizawa, J. Y. Hsu, K. Tanaka, L. González-González, A. Broto, E. Querol, M. Lluch-Senar, C. Piñero-Lambea, L. Sun, P. D. Weyman, K. S. Wise, C. Merryman, G. Tse, A. J. Moore, C. A. Hutchison III, H. O. Smith, M. Tomita, J. C. Venter, J. I. Glass, J. Piñol, Y. Suzuki, Tuning gene activity by inducible and targeted regulation of gene expression in minimal bacterial cells. *ACS Synth Biol* **7**, 1538–1552 (2018).

22. J. Salje, F. van den Ent, P. de Boer, J. Lowe, Direct membrane binding by bacterial actin MreB. *Mol. Cell* **43**, 478–487 (2011).

23. F. van den Ent, T. Izore, T. A. Bharat, C. M. Johnson, J. Lowe, Bacterial actin MreB forms antiparallel double filaments. *eLife* **3**, e02634 (2014).

24. D. Popp, A. Narita, K. Maeda, T. Fujisawa, U. Ghoshdastider, M. Iwasa, Y. Maéda, R. C. Robinson, Filament structure, organization, and dynamics in MreB sheets. *J. Biol. Chem.* **285**, 15858–15865 (2010).

25. H. Shi, B. P. Bratton, Z. Gitai, K. C. Huang, How to build a bacterial cell: MreB as the foreman of *E. coli* construction. *Cell* **172**, 1294–1305 (2018).

26. D. Takahashi, I. Fujiwara, Y. Sasajima, A. Narita, K. Imada, M. Miyata, Structure and polymerization dynamics of bacterial actin MreB3 and MreB5 involved in *Spiroplasma* swimming. *Open Biol.* **12**, 220083 (2022).

27. S. Harne, P. Gayathri, L. Beven, Exploring *Spiroplasma* biology: Opportunities and challenges. *Front. Microbiol.* **11**, 589279 (2020).

28. F. Masson, X. Pierrat, B. Lemaitre, A. Persat, The wall-less bacterium *Spiroplasma poulsonii* builds a polymeric cytoskeleton composed of interacting MreB isoforms. *iScience* **24**, 103458 (2021).

29. N. R. Martin, E. Blackman, B. P. Bratton, K. J. Chase, T. M. Bartlett, Z. Gitai, CrvA and CrvB form a curvature-inducing module sufficient to induce cell-shape complexity in Gram-negative bacteria. *Nat. Microbiol.* **6**, 910–920 (2021).

30. N. Terahara, I. Tulum, M. Miyata, Transformation of crustacean pathogenic bacterium *Spiroplasma eriocheiris* and expression of yellow fluorescent protein. *Biochem. Biophys. Res. Commun.* **487**, 488–493 (2017).

31. B. J. Karas, K. S. Wise, L. Sun, J. C. Venter, J. I. Glass, C. A. Hutchison, H. O. Smith, Y. Suzuki, Rescue of mutant fitness defects using in vitro reconstituted designer transposons in *Mycoplasma mycoides*. *Front. Microbiol.* **5**, 369 (2014).

32. T. Kasai, D. Nakane, H. Ishida, H. Ando, M. Kiso, M. Miyata, Role of binding in *Mycoplasma mobile* and *Mycoplasma pneumoniae* gliding analyzed through inhibition by synthesized sialylated compounds. *J. Bacteriol.* **195**, 429–435 (2013).

33. D. Nakane, M. Miyata, *Mycoplasma mobile* cells elongated by detergent and their pivoting movements in gliding. *J. Bacteriol.* **194**, 122–130 (2012).

34. Y. Hiratsuka, M. Miyata, T. Q. Uyeda, Living microtransporter by uni-directional gliding of *Mycoplasma* along microtracks. *Biochem. Biophys. Res. Commun.* **331**, 318–324 (2005).

35. T. Toyonaga, T. Kato, A. Kawamoto, N. Kodera, T. Hamaguchi, Y. O. Tahara, T. Ando, K. Namba, M. Miyata, Chained structure of dimeric F_1-like ATPase in *Mycoplasma mobile* gliding machinery. *mBio* **12**, e0141421 (2021).

36. Y. Kawakita, M. Kinoshita, Y. Furukawa, I. Tulum, Y. O. Tahara, E. Katayama, K. Namba, M. Miyata, Structural study of MPN387, an essential protein for gliding motility of a human-pathogenic Bacterium, *Mycoplasma pneumoniae*. *J. Bacteriol.* **198**, 2352–2359 (2016).

37. M. Nishikawa, D. Nakane, T. Toyonaga, A. Kawamoto, T. Kato, K. Namba, M. Miyata, Refined mechanism of *Mycoplasma mobile* gliding based on structure, ATPase activity, and sialic acid binding of machinery. *mBio* **10**, e02846-19 (2019).

38. J. Ye, G. Coulouris, I. Zaretskaya, I. Cutcutache, S. Rozen, T. L. Madden, Primer-BLAST: A tool to design target-specific primers for polymerase chain reaction. *BMC Bioinformatics* **13**, 134 (2012).

39. D. N. Perkins, D. J. Pappin, D. M. Creasy, J. S. Cottrell, Probability-based protein identification by searching sequence databases using mass spectrometry data. *Electrophoresis* **20**, 3551–3567 (1999).
